# Histopathology and virulence of an *in vitro*-adapted *Trypanosoma evansi* TEDC 953 strain (Thailand isolate) in mice

**DOI:** 10.14202/vetworld.2023.1008-1017

**Published:** 2023-05-13

**Authors:** Wallaya Phongphaew, Charuwan Wongsali, Thanisorn Boonyakong, Theerawat Samritwatchasai, Wissanuwat Chimnoi, Ketsarin Kamyingkird

**Affiliations:** 1 Department of Pathology, Faculty of Veterinary Medicine, Kasetsart University, Lad Yao, Chatuchak, Bangkok 10900, Thailand; 2 Veterinary Diagnostic Center, Faculty of Veterinary Medicine, Kasetsart University, Lad Yao, Chatuchak, Bangkok 10900, Thailand; 3 Laboratory Animal Unit, Research Support Center, Faculty of Veterinary Medicine, Kasetsart University, Lad Yao, Chatuchak, Bangkok 10900, Thailand; 4 Department of Parasitology, Faculty of Veterinary Medicine, Kasetsart University, Lad Yao, Chatuchak, Bangkok 10900, Thailand

**Keywords:** experimentally infected mice, histopathology, *in vitro*, *in vivo*, *Trypanosoma evansi* Thai strain, virulence

## Abstract

**Background and Aim:**

*Trypanosoma evansi* is a blood and tissue protozoan parasite affecting domestic and wild animals. The *T. evansi* Thai strain, namely, *T. evansi* from dairy cattle number 953 (TEDC 953) strain, has been successfully isolated from dairy cattle and cultivated *in vitro*. The *in vitro*-cultivated parasite is useful for biological studies, evaluation of novel chemotherapeutic agents, and production of antigens for diagnostic tests. This study aimed to observe the histopathology and virulence of an *in vitro*-adapted *T. evansi* TEDC 953 strain *in vivo*.

**Materials and Methods:**

The histopathology and virulence of the TEDC 953 strain were clarified in mice. Six mice were infected with 1 × 10^5^ trypomastigotes of TEDC 953 strain intraperitoneally, and four mice were in the negative control. Parasitemia was monitored daily, and the mice were euthanized on 30 days post-infection (DPI). Internal organs were collected for histopathological examination using hematoxylin and eosin staining.

**Results:**

Histopathological lesions were found in the liver, lung, heart, kidney, spleen, and brain of the inoculated mice. The main histopathological feature was lymphoplasmacytic inflammation in parenchyma and perivascular areas of multiple organs, and the severity of histopathological changes was related to the presence of trypomastigotes in the regional vessels. Granulomatous inflammation was seen in meninges, pleura, renal capsule, renal pelvis, and spleen of some infected mice. Four mice died at 17, 24, 26, and 27 DPI with an average parasitemia of 4.05 × 10^11^ trypomastigotes/mL. The average survival time was 23.5 DPI (mice = 4).

**Conclusion:**

This study confirmed that the TEDC 953 strain is infectious and pathogenic in mice after the continuously cultivated *in vitro*. To replace the use of experimental animals, the *in vitro*-cultivated parasite can be used instead in further studies.

## Introduction

*Trypanosoma evansi* is a flagellate protozoa parasite infecting various kinds of mammals. *Trypanosoma evansi* has only a bloodstream trypomastigote in its life cycle and is transmitted through mechanical vectors causing trypanosomiasis, also known as “surra.” It is highly pathogenic in susceptible hosts such as dogs, horses, camels, and buffaloes [1–4].

In general, *T. evansi* trypomastigotes isolated from natural-infected blood have been used in infective, virulence, pathological, and histopathological studies by infecting experimental animals such as mice [5, 6], cats [7], dogs [8], goats [9], and buffaloes [10]. Clinicopathological findings of *T. evansi* experimentally infected in water buffalo (*Bubalus bubalis*) show that *T. evansi* is both an intravascular and extravascular parasite [10]. Histopathological study of *T. evansi* infection in albino mice has indicated enlargement of the spleen with multinucleated giant cell formation, petechial hemorrhage in the liver with vacuolar degeneration to coagulative necrosis, edema, congestion, and mild inflammatory lungs, a mildly degenerative brain, tubular degeneration of the kidneys, and interstitial edema of the heart [5]. In mice, the symptoms of *T. evansi* infection are similar to those in horses [6]. Interestingly, the highly pathogenic *T. evansi* strains have not led to significant pathological lesions when compared with the less pathogenic strain that showed significant pathological changes in the spleen with severe-suppurative encephalitis and cholangiohepatitis [6]. Conversely, seven different *T. evansi* strains isolated from various hosts have been evaluated in the mouse model, which indicated the variation of the pathogenicity of *T. evansi* infection [11]. Similarly, a variation in pathogenicity between buffalo and dog *T. evansi* isolates infection in mice indicated the presence of trypomastigotes in mice internal organs; however, no trypomastigotes were found in mice blood vessels infected with the *T. evansi* isolated from dog [12].

Previously, *in vitro* cultivation of the *T. evansi* Thai strain, namely, the TEDC 953 strain was successfully developed. This strain was isolated from dairy cattle in Lampang province, Thailand in 2017 for the development of *in vitro* cultivation. This *in vitro*-cultivated parasite can be useful for antigen production and further studies. The *in vitro*-cultivated pathogen has been known to reduce its virulence and pathogenicity *in vivo* after long being used in the laboratory [13]. Unlike the other studies, an *in vitro*-adapted for 100 days strain of the *T. evansi* Thai strain, namely, the “TEDC 953 strain,” was used to clarify the pathogenicity *in vivo* in this study. Parasitemia, mortality rates, and clinical symptoms were also monitored. The result from this study would support the usefulness of *in vitro*-cultivated parasites isolated in Thailand on diagnostic test development, trypanocidal development, prevention, and control of trypanosomiasis. The isolation and evaluation of local trypanosome strains are very important to analyze the risk of infection and endemicity and also estimate the economic loss by their infection. This study is also valuable for revealing the pathogenicity of local trypanosome strains.

This study aimed to observe the histopathology and virulence of an *in vitro*-adapted *T. evansi* TEDC 953 strain *in viv*o.

## Materials and Methods

### Ethical approval

The animal study protocol was approved by the Kasetsart University Animal Ethics Committee (Approval no. ACKU65-VET-009).

### Study period and location

This study was conducted from March 3, 2022 to April 4, 2022 at the Faculty of Veterinary Medicine, Bangkok, Thailand.

### Parasite strain and mouse inoculation

The *in vitro*-adapted trypomastigotes of *T. evansi*, namely, the TEDC 953 strain, were used for mouse inoculation by 1 × 10^5^ trypomastigotes/mice as indicated by Krishnamoorthy *et al*. [12]. The infected group consisted of six mice, and the non-infected group consisted of four mice as described by Kamidi *et al*. [14]. TEDC 953 strain was collected after being cultivated *in vitro* for 100 days. The collected parasites were centrifuged twice at 11,200× *g*, 4°C for 5 min, and suspended with chilled and sterile 1% glucose in phosphate saline solution (PSG). Trypomastigotes were observed under a microscope to confirm their movement and viability. Trypomastigotes were counted using a counting chamber before inoculation. Two hundred microliters of PSG containing TEDC 953 strain trypomastigotes were injected in six mice intraperitoneally as a study group. Two hundred microliters of PSG alone were injected intraperitoneally in four mice as a negative control group. All mice used in this study were 6-week-old female ICR mice (purchased from National Laboratory Animals Center, Thailand).

### Estimation of parasitemia *in vivo*

The presence of live parasites was estimated daily from each mouse using a direct blood smear method. Briefly, approximately 2 μL of blood was collected from the mouse tail tip and measured using a micropipette. Collected fresh blood was immediately placed on the glass slide, covered with a 22 × 22 mm coverslip, and observed under the phase-contrast microscope (Olympus, Japan). Total blood areas were counted under 200× magnification, and the number of live trypomastigotes was counted for three sets under the same magnification. Parasitemia was calculated in the unit of trypomastigotes/mL.

### Evaluation of *T. evansi* virulence *in vivo*

Evaluation of TEDC 953 strain virulence in mice, parasitemia, was monitored daily for 30 days. Briefly, 2 μL of the blood was collected from the mice’s tail tip, trypomastigotes were counted, and the number of trypomastigotes/mL was calculated. Clinical symptoms were also observed daily. The mortality rate was calculated using a formula: (Number of dead mice/number of total infected mice) × 100. The category of *T. evansi* virulence was evaluated as previously described by Kamidi *et al*. [14].

### Euthanasia, collection of specimens, and histopathological examination

Mice were euthanized on the 30 days post-infection (DPI) using the protocol as previously described by Kamidi *et al*. [14]. Visceral organs including the brain, heart, lungs, liver, stomach, spleen, intestine, and kidney were collected and stored in 10% formalin. All specimens were proceeded for formalin-fixed, paraffin-embedded tissues, and stained with hematoxylin and eosin as previously described by Drury and Wallington [15]. All histopathological specimens were examined under a light microscope by a Thai board-certified pathologist.

## Results

### Parasitemia and virulence of TEDC 953 strain in mice

**Figure-1:**
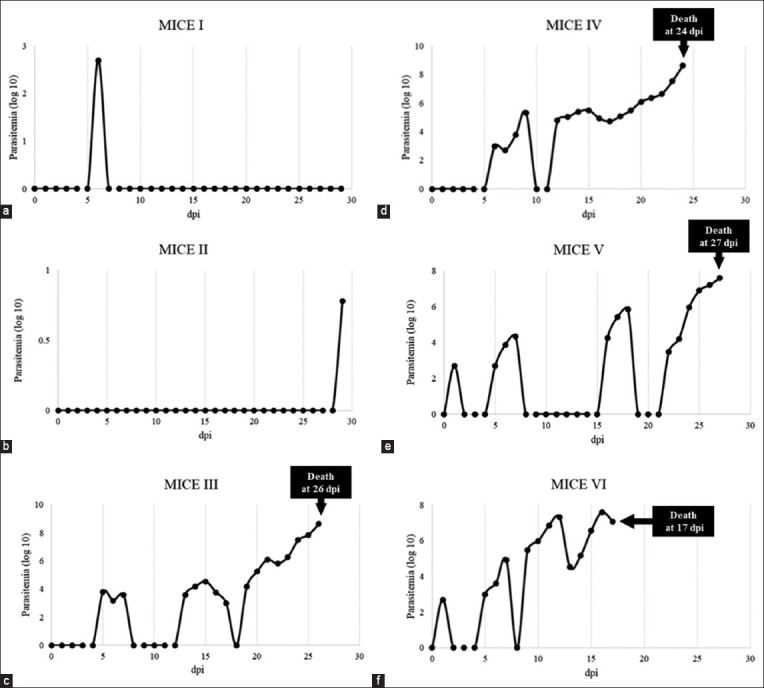
Estimated parasitemia of *Trypanosoma evansi* TEDC 953 strain-infected mice I-VI (a-f) (mortality rate was partially published by Kamyingkird *et al*. [13]).

Live trypomastigotes were detected in the infected mice blood from day one pi. Estimated parasitemia from four infected mice was gradually increased and reached 1 × 10^9^ trypomastigotes/mL from 17, 24, 26, and 27 DPI, with a fluctuating pattern (Figure-1) [13]. The survival rate of mice infected with the *T. evansi* TEDC 953 strain falls between 11 and 30 days which are categorized as a moderate virulence strain. An average survival time of 23.5 DPI was calculated from four dead mice. However, the other two infected mice survived for more than 30 DPI, which is categorized as low virulence.

### Histopathological findings through *in vitro*-cultivated *T. evansi* TEDC 953 strain

Histopathological findings of non-infected mice were described in Table-1. Lymphoid enlargement and hyperplasia were found in the spleen of two mice and mild perivascular lymphoplasmacytic infiltration of the kidney in one mouse (Table-2). Extramedullary hematopoiesis (EMH) was found in the spleen of all mice, both non-inoculated and inoculated mice.

**Table-1:** Histopathological findings in non-infected mice (n = 4).

Organs	Mice I (30 DPI)	Mice II (30 DPI)	Mice III (30 DPI)	Mice IV (30 DPI)
Brain	Normal	Normal	Normal	Normal
Heart	Normal	Normal	Normal	Normal
Lung	Normal	Normal	Normal	Normal
Liver	Normal	Normal	Normal	Normal
Spleen	EMH	Lymphoid hyperplasia and enlargement, EMH	EMH	Lymphoid hyperplasia and enlargement, EMH
Kidney	Normal	Mild perivascular lymphoplasmacytic infiltration	Normal	Normal

EMH=Extramedullary hematopoiesis

**Table-2:** Histopathological findings of the specimens in TEDC 953 strain-infected mice (n = 6).

Organs	Mice I (30 DPI)	Mice II (30 DPI)	Mice III (died at 26 DPI)	Mice IV (died at 24 DPI)	Mice V (died at 27 DPI)	Mice VI (died at 17 DPI)
Brain	Mild perivascular lymphocytic meningitis	Mild lymphocytic meningitis and focal mineralization	Focal granulomatous meningitis and perivascular lymphocytic meningitis	Perivascular lymphocytic meningitis	Mild perivascular lymphocytic meningitis	Lymphocytic perivascular cuffing and mild perivascular lymphocytic meningitis
Heart	Normal	Normal	Mild lymphoplasmacytic epicarditis and myocarditis	Mild lymphoplasmacytic epicarditis	Mild lymphoplasmacytic myocarditis	Normal
Lung	Pulmonary multifocal hemorrhage, mild interstitial pneumonia, pulmonary congestion, and micro thrombosis	Moderate interstitial pneumonia	Interstitial pneumonia, pulmonary congestion, edema, and lymphoplasmacytic bronchitis	Severe pulmonary congestion and edema	Severe interstitial pneumonia	Moderate interstitial pneumonia, moderate pulmonary congestion, and mild pulmonary edema with microthrombosis
Liver	Normal	Mild perivascular and periportal lymphoplasmacytic infiltration	Severe, diffuse, lymphoplasmacytic and perivascular, periportal hepatitis, and multifocal necrosis of hepatocytes	Severe, diffuse, lymphoplasmacytic and perivascular, periportal hepatitis	Moderate lymphoplasmacytic perivascular and periportal hepatitis, and multifocal necrosis of hepatocytes	Severe, diffuse, lymphoplasmacytic and perivascular, periportal hepatitis, and multifocal necrosis of hepatocytes
Spleen, lymph node	Lymphoid hyperplasia and enlargement, mild hemosiderosis, and EMH	Lymphoid hyperplasia and EMH	Granulomatous lymphadenitis, lymphoid hyperplasia, and EMH	Lymphoid depletion and necrosis, EMH	Lymphoid depletion and necrosis, EMH	Lymphoid depletion and necrosis, multifocal hemorrhage, and EMH
Kidney	Mild lymphoplasmacytic pyelitis and acute tubulonephrosis	Mild perivascular lymphoplasmacytic infiltration and acute tubulonephrosis	Mild lymphoplasmacytic interstitial nephritis and acute tubulonephrosis, renal capsular granulomatous inflammation, and lymphoplasmacytic pyelitis	Acute tubulonephrosis	Severe lymphoplasmacytic pyelitis and acute tubulonephrosis	Acute tubulonephrosis

EMH=Extramedullary hematopoiesis

While in the TEDC 953-inoculated mice, histopathological lesions were seen in several organs, including the liver, lung, heart, kidney, spleen, and brain of the TEDC 953 strain-inoculated mice. The major histopathological finding was perivascular infiltration of lymphocytes and plasma cells with or without the presence of trypomastigotes in the vascular lumens.

#### Brain

**Figure-2:**
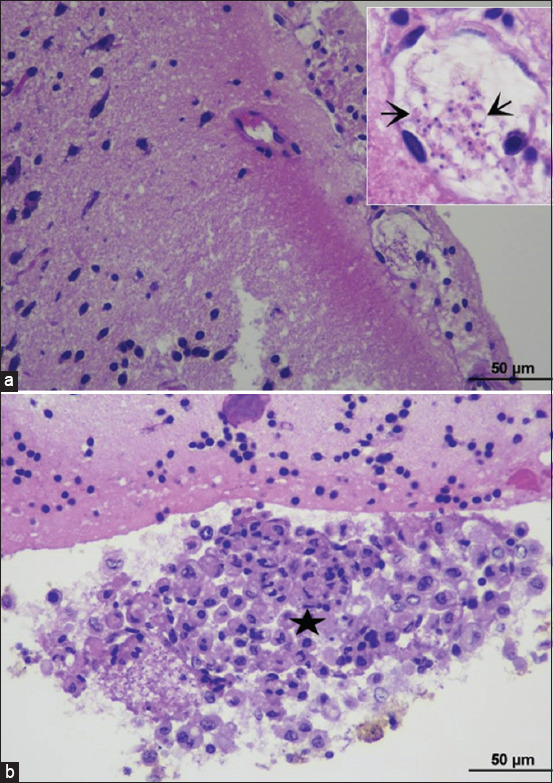
Histopathological findings of the brain in TEDC 953 infected mice; the presence of aggregated trypomastigotes in cerebral vessels, black arrow (a), granulomatous meningitis, characterized by accumulation of phagocytic macrophages, black star (b), 400× magnification.

The brain sections of the TEDC 953 strain-inoculated mice revealed a mild infiltration of lymphocytes into the meninges, with several trypomastigotes in the meningeal and cerebral vessels. Microscopically, one inoculated mouse developed focal granulomatous meningitis (Figure-2 and Table-2).

#### Heart

**Figure-3:**
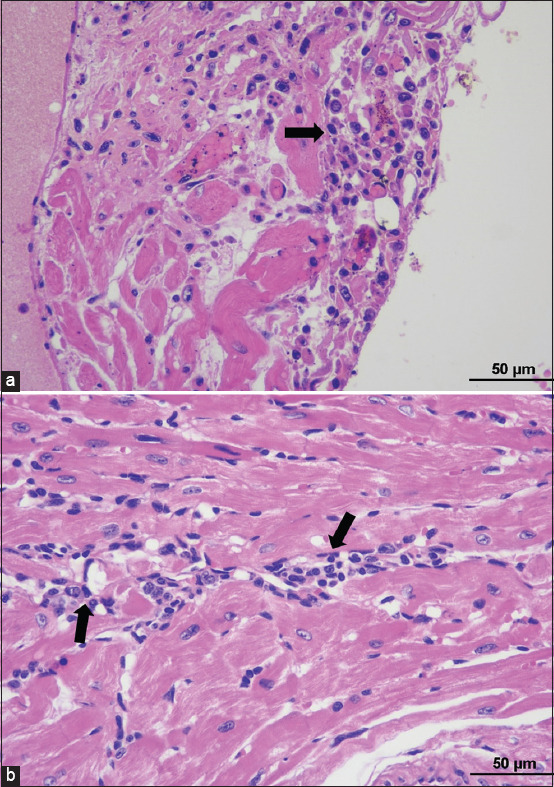
Histopathological findings of heart in TEDC 953 infected mice; lymphoplasmacytic infiltration into epicardium, black arrow (a), lymphoplasmacytic perivascular myocarditis, black arrow (b), 200× magnification.

Half of the inoculated mice (3/6) had mild lymphoplasmacytic and fewer granulocyte infiltration into the epicardium and myocardium with focal myocardial necrosis. Aggregation of lymphocytes and plasma cells was also noticed in the surrounding fat, and inflammation was especially noted in the perivascular areas (Figure-3 and Table-2).

#### Lung

**Figure-4:**
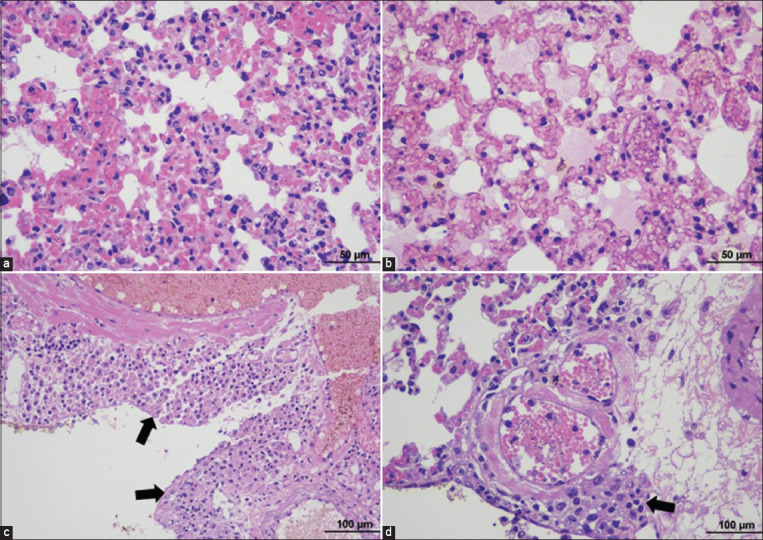
Histopathological findings of the lung in TEDC 953 infected mice; interstitial pneumonia, characterized by thickening of the alveolar wall and infiltration of lymphocytes and presence of trypomastigotes in alveolar capillaries (a), 400× magnification. Pulmonary congestion and edema, characterized by dilated alveolar capillaries with congested blood and accumulation of homogenous eosinophilic edematous fluid in the alveolar lumens (b), 400× magnification. Granulomatous pleuritis, visceral pleura was thickened due to accumulation of macrophages, black arrow (c), 600× magnification and lymphoplasmacytic perivascular cuffing in lung, black arrow (d), 600× magnification.

Histopathological findings of lung sections were interstitial pneumonia, characterized by thickening of the alveolar wall due to infiltration of mononuclear inflammatory cells. Pulmonary congestion and edema were also detected, and the alveolar capillaries were dilated with congested blood. Homogenous eosinophilic edematous fluid was noted with the presence of a moderate number of hemosiderin-laden macrophages in the alveolar lumens. Trypomastigotes were seen in the alveolar capillaries. Some infected mice demonstrated granulomatous pleuritis and also infiltration of mononuclear inflammatory cells around vessels (Figure-4 and Table-2).

#### Liver

**Figure-5:**
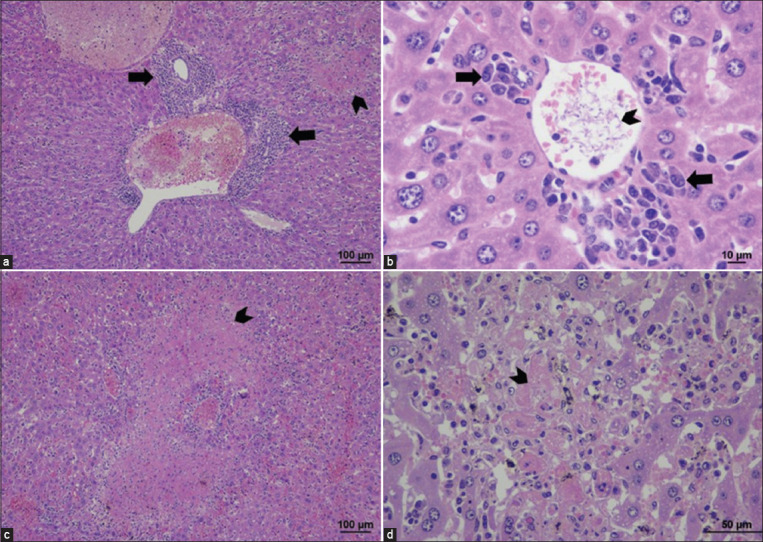
Histopathological findings of the liver in TEDC 953-inoculated mice; lymphoplasmacytic infiltration at the periportal area, black arrow (a), 100× magnification, presence of bloodstream form trypomastigotes in central vein (arrowhead) with extramedullary hematopoiesis around the central vein (black arrow) (b), 600× magnification, focal coagulative necrosis of hepatocytes (arrowhead), and periportal lymphoplasmacytic infiltration (c), 400× magnification, and coagulative necrosis of hepatocytes (arrowhead) with infiltration of mononuclear inflammatory cells (d), 400× magnification.

All liver specimens of infected mice showed histopathological changes (6/6). The histopathological examination demonstrated distorted hepatic cords and diffusely swollen hepatocytes. The sinusoids were dilated due to congested blood. Infiltration of lymphocytes and plasma cells was detected in the perivascular, periportal areas, and some areas of hepatic parenchyma. The severity of inflammation ranged from mild-to-severe. Numerous trypomastigotes were found in the affected vessels or sinusoids, and multifocal coagulative necrosis of hepatocytes was seen in the severely infected mice (Figure-5 and Table-2).

#### Kidney

**Figure-6:**
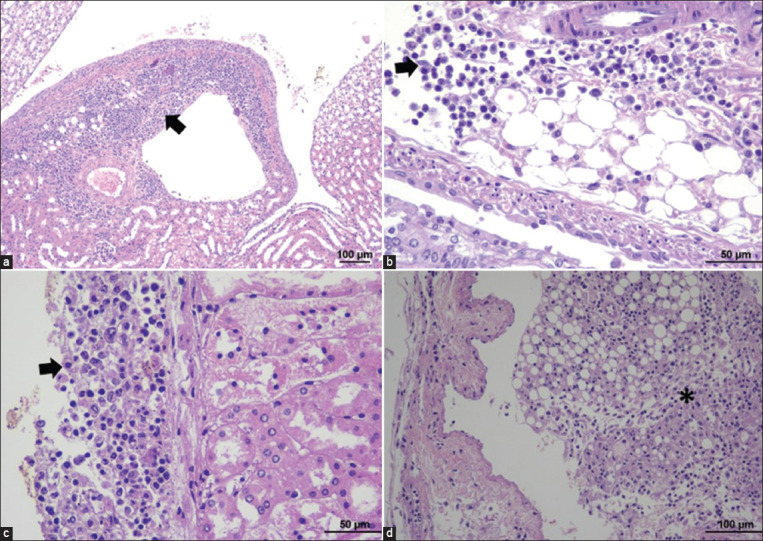
Histopathological findings of the kidney in TEDC 953-inoculated mice; lymphoplasmacytic pyelitis (black arrow) (a), 100× magnification, infiltration of lymphocytes and plasma cells into the renal pelvis (black arrow) (b), 400× magnification, granulomatous inflammation at renal capsule (black arrow) (c), 400× magnification and granulomatous pyelitis (asterisk) (d), 200× magnification.

According to the histopathological examination of the kidneys of inoculated mice, four of six inoculated mice showed perivascular and interstitial infiltration of lymphocytes and plasma cells. The inflammation was also presented at the renal pelvis. Alterations of the renal tubular epithelium of proximal and distal convoluted tubules were detected. The cells were swollen and partially obliterated the tubular lumens. The cytoplasm had increased the eosinophilia, and the nuclei were changed. These alterations indicated acute tubulonephrosis. Some hyaline droplets were detected in the renal tubules. Glomeruli were shrunken and degenerated. Granulomatous inflammation at the renal capsule and renal pelvis was seen in two inoculated mice (Figure-6 and Table-2).

#### Spleen

**Figure-7:**
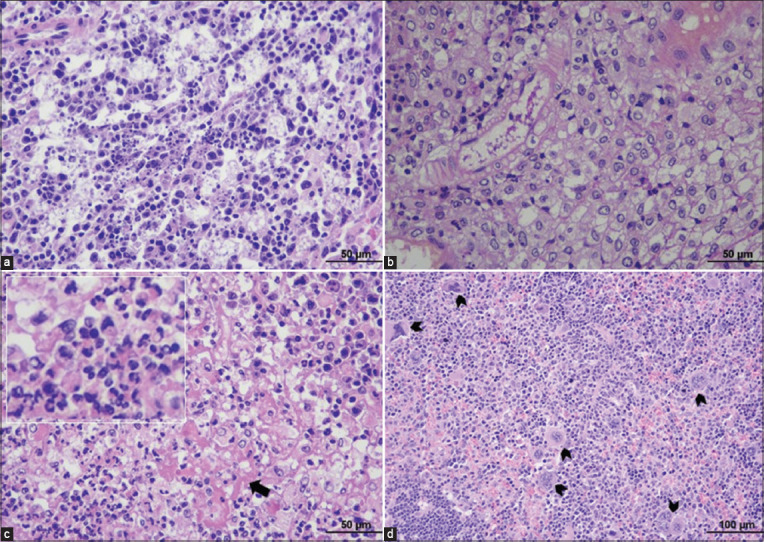
Histopathological findings of the spleen in TEDC 953-inoculated mice; necrosis of lymphoid cells (a), 200× magnification, granuloma formation in the splenic parenchyma (b), 400× magnification, focal necrosis (black arrow) with infiltration of neutrophils (c), 400× magnification and presence of numerous megakaryocytes (arrowhead) indicating increased extramedullary hematopoiesis (d), 200× magnification.

Microscopically, the spleen demonstrated enlargement of white pulps with hyperplasia of immature lymphoid cells in the three inoculated mice (3/6). Granulomatous lymphadenitis was found in one of those three mice, whereas lymphoid depletion and necrosis of white pulps with numerous tingible body macrophages were noticeable in another half of inoculated mice (3/6). Hemorrhage and hemosiderosis were detected in one mouse. The presence of many megakaryocytes in red pulps, indicating EMH, was seen in all spleen sections of both control and inoculated mice. This phenomenon was considered a common feature found in rodents. However, the number of megakaryocytes detected in infected mice was relatively higher than that in non-inoculated mice (Figure-7 and Table-2).

## Discussion

This study has used *in vitro*-adapted *T. evansi* for histopathological demonstration for the first time. Previously, it has been known that *T. evansi* infection would be severe and cause death in mice due to the susceptibility of the mice to the infection [4]. A negative immune response is responsible for the negative effect on the infected animals [7].

In our results, all degrees of histopathological changes were detected in the TEDC 953 strain-inoculated mice in multiple organs, including the brain, heart, lung, liver, kidneys, and spleen. The findings were correlated with the presence of trypomastigotes in the blood vessels, which indicated the parasitemia stage, especially in the TEDC 953 strain-inoculated mice III–VI that exhibited high parasitemia and died within 30 DPI. *Trypanosoma evansi*, a salivarian species, is the only species that present the “trypomastigote” stage or “bloodstream form” in mammalian blood circulation [4]. Once the live and motile parasites invade the host, they seek nutrients to survive. Therefore, trypomastigotes can be detected in host blood vessels. *Trypanosoma evansi* do not infect insect epithelial midgut due to the lack of kinetoplast DNA in the invertebrate host, in which the parasites require to develop into the next stage [4]. Therefore, the presence of the trypomastigotes of *T. evansi* in the blood vessels can normally be found and is known as “the hemolymphatic stage I” [16]. In addition, trypanosomes feed by absorbing nutrients, including glucose, amino acids, and oxygen, through their outer membrane to generate energy to perform vital processes in the blood circulation, tissue fluid, or blood plasma of the host [17]. In the brain, perivascular meningitis was found in all TEDC 953-inoculated mice but not in the non-infected mice. In “the meningoencephalitis stage II,” the parasites cross the blood–brain barrier and infect the central nervous system, causing neurological symptoms [18]. Macrophage and perivascular inflammatory cell infiltration occurs in the leptomeninges and white matter [16]. Correspondingly, some infected mice in the present study developed lymphocytic perivascular cuffing and granulomatous meningitis. In the heart, mild lymphoplasmacytic myocarditis was observed in three TEDC 953 strain-inoculated mice. However, three normal hearts were also observed. The findings of this study indicate inflammatory cell infiltration in the epicardium, myocardium, surrounding fats, and perivascular areas. Myocarditis is a major mortality cause and commonly occurs in African trypanosome infections [19]. The direct invasion of the parasites into the myocardium causes cardiac damage alongside inducing autoimmune mechanisms [19]. In the lungs, abnormality was indicated from mild pneumonia to severe pulmonary congestion in six TEDC 953 strain-inoculated mice. In this situation, trypomastigotes in the alveolar arteries clearly induced inflammatory cells, such as lymphocytes, eosinophils, and macrophages, to infiltrate the lungs and cause the thickening of the alveolar wall, pulmonary congestion, edema, pleuritis, and perivascular cuffing. This finding is also concordant with those of a previous report Nieman *et al*. [19]. In the liver, mild-to-severe perivascular periportal hepatitis was observed in five TEDC 953 strain-inoculated mice. Particularly, in mice III, IV, and VI, which were severely infected, multifocal necrosis of hepatocytes was clearly detected. Trypanosomes were abundant in the hepatic vessels. Moreover, organ damages were clearly observed in the spleen and kidneys in all the TEDC 953 strain-inoculated mice.

The predominant histopathological features observed in our study were lymphoplasmacytic inflammation in the parenchyma and perivascular areas of multiple organs, and the severity of the histopathological changes was associated with the presence of trypomastigotes in the regional vessels. Furthermore, granulomatous inflammation was observed in the meninges, pleura, renal capsule, renal pelvis, and spleen of some infected mice. The presence of granulomatous inflammation indicated the progression of the disease, which was induced by inflammatory responses [20]. Disease progression might also have depended on the individual immune response and variation of parasitemia levels [21]. The presence of the parasite not only induced inflammatory cell infiltration but also produced various surface glycoproteins in the infected portion of the organs that can lead to the production of pyruvates involved in acidosis, free radicals related to oxidative stress, inflammatory cytokine and chemokine induction, and hepatic cell dysfunctions [22]. Furthermore, there was evidence of increased EMH, which is the formation and activation of blood cells outside the bone marrow but inside the organ, including the liver and spleen, indicating a response to hematopoietic stress in the infected mice. Extramedullary hematopoiesis occurs when there is severe loss of red blood cells [23]. However, the EMH in the non-infected mice was considered normal microscopic appearance of the spleen of rodents, especially young rodents [24]. The findings of the present study suggest that the EMH was notably high in the *T. evansi*-infected mice to respond to anemia, which is consistent with the consequence of trypanosomiasis reported in a previous study Marins-Dos-Santos *et al*. [25]. Extramedullary hematopoiesis was detected in both the spleen and liver of the infected mice, whereas normal mice exhibited EMH only in the spleen. In addition, we noted emaciation of the TEDC 953 strain-inoculated mice; however, this observation was not included in the results, as it has already been described elsewhere Bal *et al*. [5], Aquino *et al*. [8], Krishnamoorthy *et al*. [12], Kamidi *et al*. [14].

Pathological changes in the vital organs may also be caused by a combination of oxidative stress [26], peroxides, and free radicals released during infection [22] and hypoglycemia [19, 27]. Weight loss and packed cell volume can significantly decrease in *T. evansi*-infected animals, as previously described in several studies [4, 5, 12, 14]. *Trypanosoma evansi*-infected animals exhibited erythrocyte abnormalities, adhesion of the parasite to the red blood cells, and erythrophagocytosis, which were the causes of anemia [28]. *Trypanosoma evansi* can generate adenosine triphosphate from glucose catabolism, produce free radicals, induce oxidative stress, be involved with lipid peroxidation of erythrocytes, and cause membrane injury, osmotic fragility, and red blood cell destruction [22]. However, anemia was not evaluated in this study as it has already been confirmed previously Bal *et al*. [5], Krishnamoorthy *et al*. [12], Kamidi *et al*. [14]. Herein, the histopathological changes were consistent with those reported by Bal *et al*. [5], Sawitri and Damayanti [6], Perrone *et al*. [11], Kamidi *et al*. [14]. Our results suggest that the *in vitro*-adapted *T. evansi* induced clinical symptoms and histological alterations in the experimental animals. We demonstrated that the TEDC 953 strain caused parasitemia, morbidity, and mortality in laboratory mice. Thus, according to the survival rate of 30 days within inoculation, the TEDC 953 strain was considered a moderate-to-low virulence strain.

## Conclusion

The *in vitro*-cultivated TEDC 953 strain can maintain its pathogenicity. This *in vitro*-adapted *T. evansi* would be useful for further therapeutic and diagnostic tool development.

## Authors’ Contributions

WP: Histopathological examination and drafted the manuscript. CW: Histopathological preparation. TB: Technical assistance in the experimental animal laboratory. TS: Facilitated technical assistance in the experimental animal laboratory. WC: Performed euthanasia and specimen collection. KK: Designed, conceptualized, and performed *in vitro* and *in vivo* experiments and prepared the manuscript. All authors have read, reviewed, and approved the final manuscript.
